# Superimposed Segmental Juvenile Dermatomyositis: A Case Report

**DOI:** 10.7759/cureus.94257

**Published:** 2025-10-09

**Authors:** Constanza García-Lopez, Kevin Hernández-Lara, Luz Orozco-Covarrubias, Pedro Pasquel-García Velarde, Marimar Saez de Ocariz

**Affiliations:** 1 Department of Pediatrics, National Institute of Pediatrics, Mexico City, MEX; 2 Department of Dermatology, National Institute of Pediatrics, Mexico City, MEX; 3 Department of Pathology, National Institute of Pediatrics, Mexico City, MEX

**Keywords:** blaschko lines, cutaneous mosaicism, juvenile dermatomyositis, loss of heterozygosity, segmental distribution, superimposed

## Abstract

Superimposed segmental juvenile dermatomyositis (SSJDM) is a rare and underrecognized variant of juvenile dermatomyositis (JDM) characterized by an initial, long-standing, well-defined lesion following Blaschko lines, later accompanied by classic cutaneous and systemic features. SSJDM likely results from early developmental genetic mutations causing loss of heterozygosity and type 2 cutaneous mosaicism. We describe an eight-year-old girl with morpheiform SSJDM and review the previously reported cases, analyzing clinical features, treatment, and outcomes. We also propose a clinical classification for the segmental presentations, which include morpheiform, linear calcinosis, lichenoid plaques, and erythematous-scaly plaques. This report aims to improve recognition of atypical segmental lesions to enable earlier diagnosis and treatment of JDM.

## Introduction

Juvenile dermatomyositis (JDM) is a rare and potentially fatal autoimmune disease that primarily affects the skin and muscles. The cutaneous features are key in differentiating JDM from other inflammatory myopathies and often precede the onset of muscle weakness [1].

Recently, some cases of JDM presenting with segmental Blaschko-linear lesions, later followed by the classic cutaneous manifestations, have been described as superimposed segmental juvenile dermatomyositis (SSJDM) [2]. SSJDM is thought to result from type 2 cutaneous mosaicism (homozygous segments within heterozygous skin) or superimposed lesions (segmental lesions coexisting with nonsegmental ones) [3,4].

SSJDM is characterized by an initial, long-standing, well-defined lesion following Blaschko lines, subsequently accompanied by classic cutaneous signs and systemic manifestations such as muscle weakness, fatigue, and laboratory abnormalities (e.g., elevated muscle enzymes and abnormal electromyography) [2]. This rare variant, likely underdiagnosed, has so far been described only in pediatric patients and exhibits heterogeneous clinical features. Here, we report the case of a female patient with SSJDM, review the currently published cases, and propose a clinical classification of the lesions.

## Case presentation

**Figure 1:**
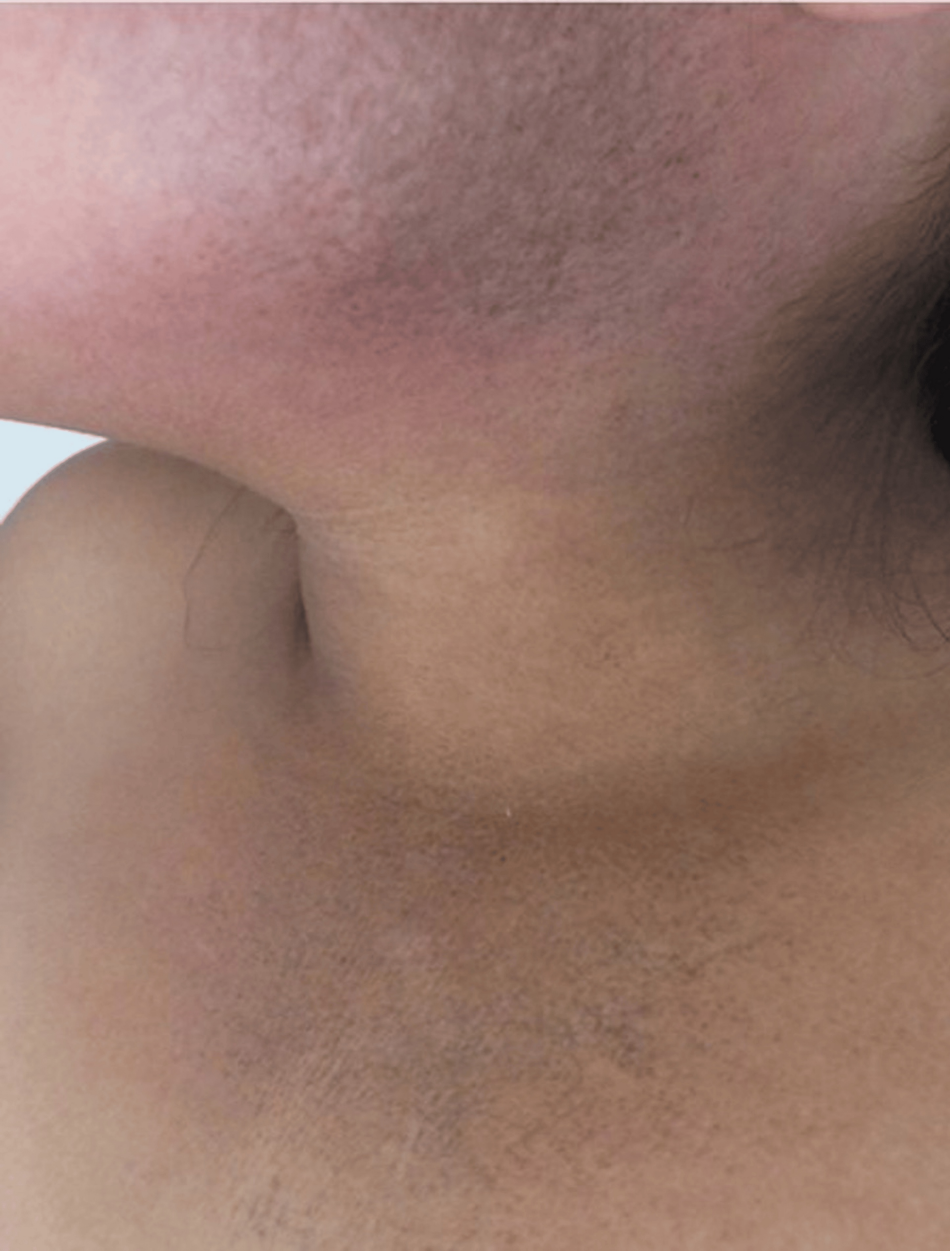
Poikiloderma and V sign The erythema, hyperpigmentation, and slight atrophy appear on the cheek, and the V sign appears on the chest

**Figure 2:**
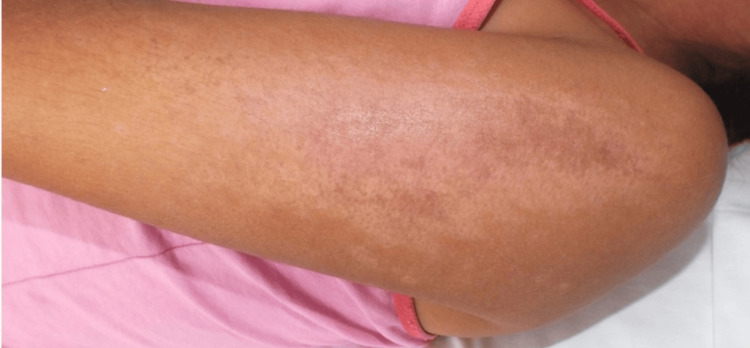
Superimposed morpheiform plaque An ill-defined, shiny, erythematous plaque appears on the external surface of the left arm

An eight-year-old girl with no significant medical or family history presented with a two-month history of cutaneous rashes, followed by myalgias, muscle weakness, facial and lower limb edema, and high-grade fever over the preceding 10 days. Initial treatments with systemic antibiotics and nonsteroidal anti-inflammatory drugs were ineffective. Suspected systemic lupus erythematosus led to her referral for further evaluation. During the examination, the patient presented with bilateral malar erythema, a heliotrope rash, poikiloderma, and a V sign (see Figure 1). Additionally, she had Gottron papules on the knuckles, elbows, and knees. There was also a linear, ill-defined, shiny, erythematous plaque measuring 14 × 3 cm on the external surface of the left arm (see Figure 2). This plaque had appeared six months prior to the onset of the other cutaneous features. Additional findings included cervical lymphadenopathy, proximal muscle weakness (2/5), and mild headache.

**Figure 3:**
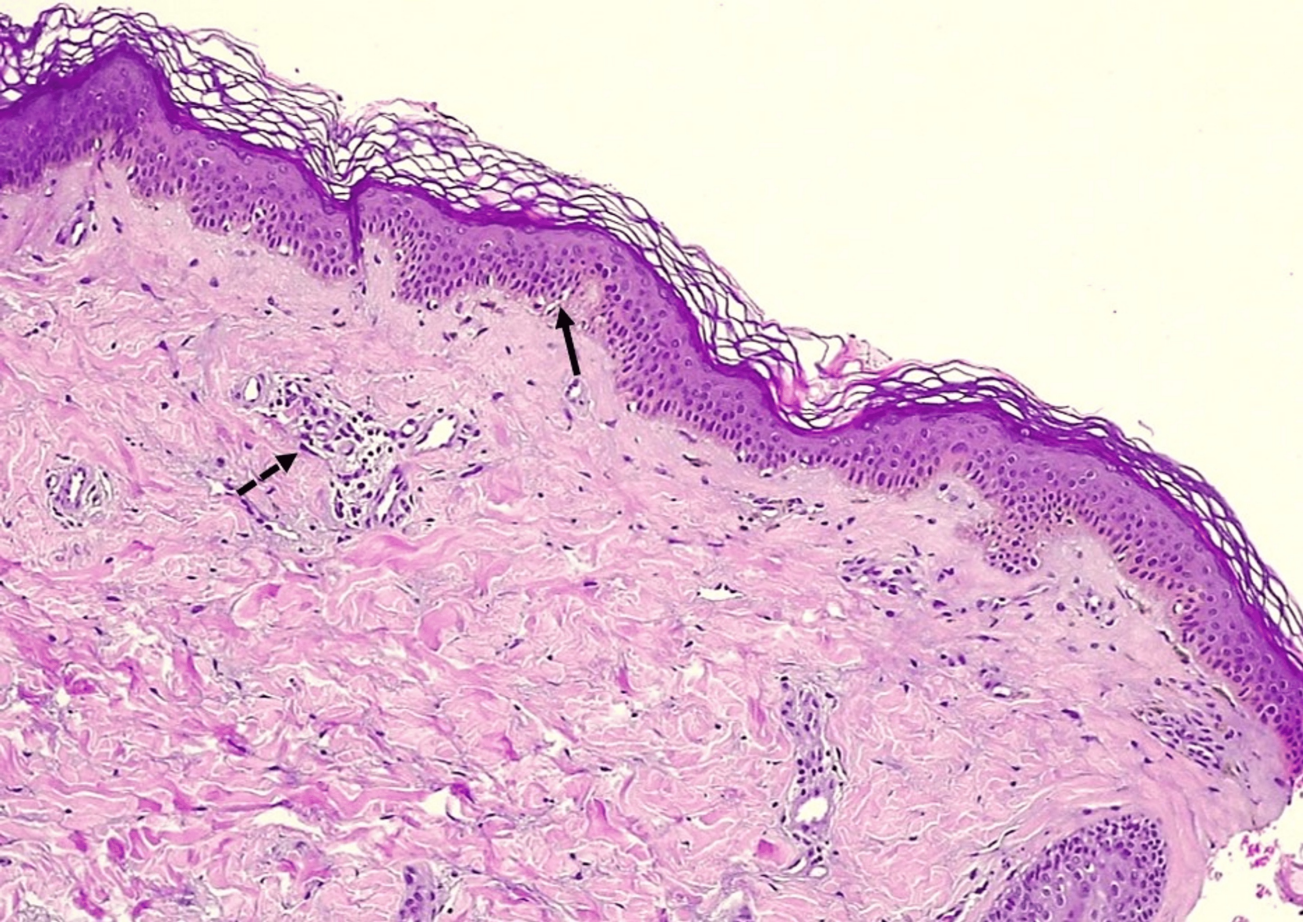
Histology of morpheiform plaque Focal basal cell vacuolation (complete black arrow) and perivascular lymphocytic infiltrate (incomplete black arrow)

**Figure 4:**
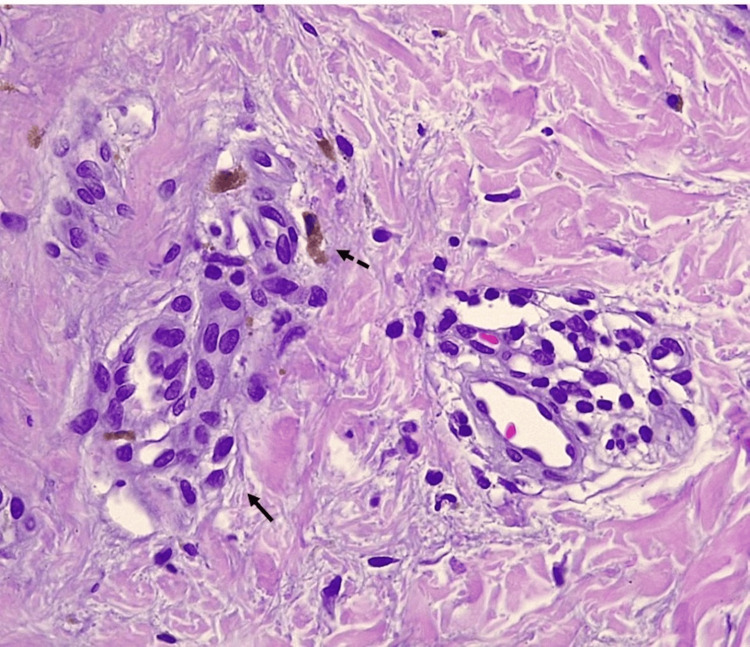
Closer view on histopathology of morpheiform plaque Perivascular lymphocytic infiltrate (complete arrow) and pigment incontinence (incomplete arrow)

With a presumptive diagnosis of JDM, a complete work-up was performed. Laboratory tests revealed elevated levels of creatine kinase, lactate dehydrogenase, aspartate aminotransferase, and alanine aminotransferase (Table 1), along with myopathic changes on electromyography. Proximal nailfold capillaroscopy revealed periungual erythema and capillary dropout. A biopsy of the atypical morphea-like linear plaque showed flattening of the interpapillary processes, focal basal cell vacuolation, pigment incontinence with melanophages, and discrete superficial perivascular lymphocytic infiltrates (Figures 3, 4).

**Table 1:** Laboratory findings

Enzyme	Patient's laboratory value	Normal laboratory values [5]
Creatine kinase	18,600 UI/L	50-231 UI/L
Lactate dehydrogenase	1,353 UI/L	192-321 UI/L
Aspartate aminotransferase	476 UI/L	18-36 UI/L
Alanine aminotransferase	450 UI/L	9-25 UI/L

**Figure 5:**
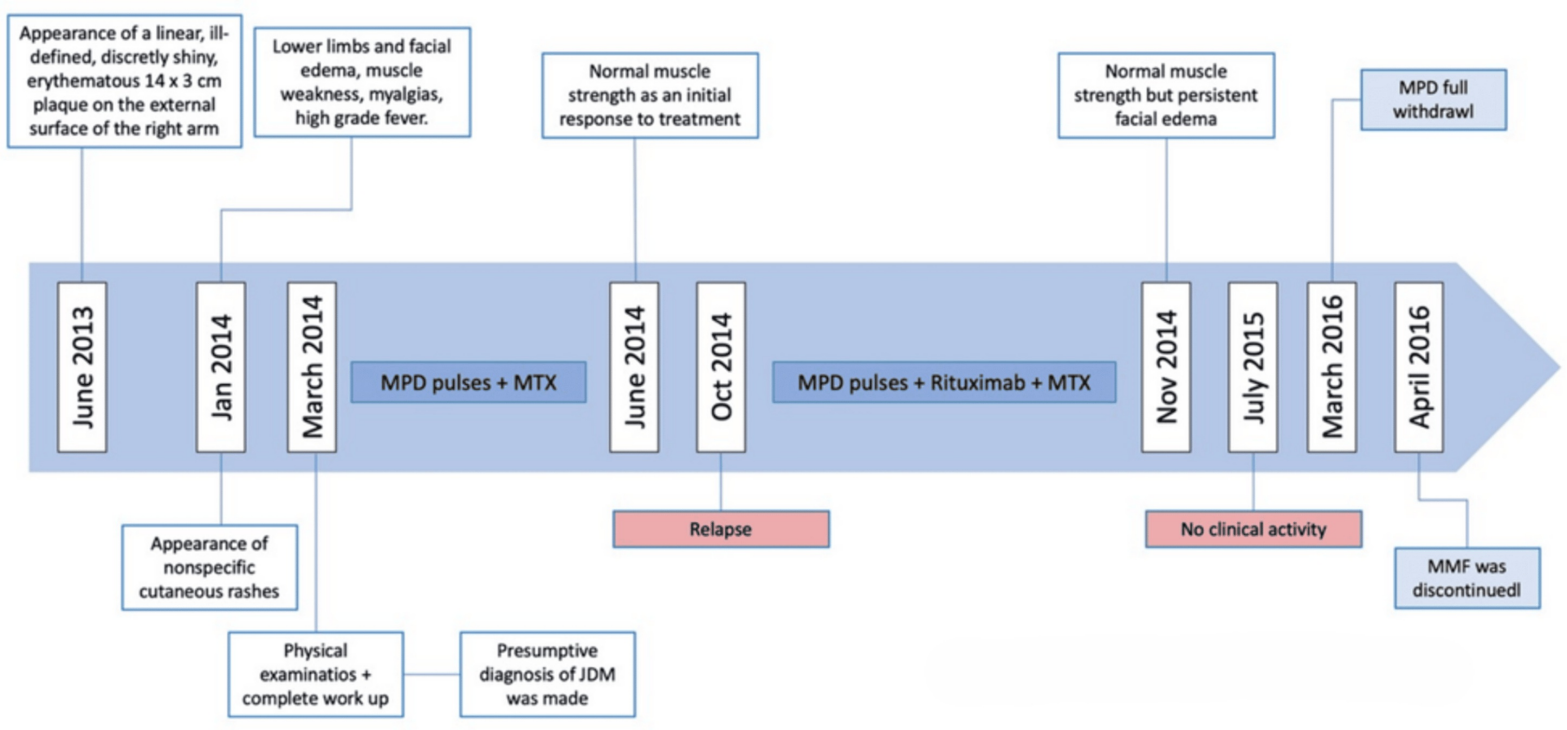
Timeline summarizing the patient's clinical course JDM: juvenile dermatomyositis; MPD: methylprednisolone; MMF: mycophenolate mofetil; MTX: methotrexate

Treatment began with three pulses of methylprednisolone, followed by oral prednisolone and methotrexate, resulting in improved muscle strength within two months. A relapse after five months was managed with three further methylprednisolone pulses and four doses of rituximab and mycophenolate mofetil, with methotrexate being discontinued. One month later, muscle strength normalized, and there was no clinical activity after eight months of therapy. Prednisolone was tapered over eight months, and mycophenolate mofetil was discontinued nine months later (Figure 5).

Search strategy and case selection

We searched PubMed, Scopus, and Latin American and Caribbean Literature on Health Sciences using the terms “superimposed” OR “segmental” OR “Blaschko-linear” AND “dermatomyositis” for articles published up to December 2024. Inclusion criteria were as follows: 1) original studies, 2) any language, 3) case reports or series, and 4) reporting segmental or Blaschko-linear initial manifestations. From each selected paper, we extracted data on age, sex, type of segmental lesion, other cutaneous features of diabetes mellitus (DM), systemic manifestations, histopathology, and treatment.

Results

A total of 11 pediatric SSJDM cases, including ours, were identified (Table 2) [1,2,4-12]. The median age at onset was nine years (range, 3-14), with a male-to-female ratio of 1.8:1. Segmental lesions preceded the classic manifestations of JDM in all patients.

**Table 2:** Cases of juvenile dermatomyositis with initial segmental lesions

Study	Sex and age at onset	Segmental lesion	Nonsegmental lesions	Muscle weakness	Histopathology of segmental lesion	Treatment	Follow-up (months) and clinical course
Boente Mdel et al. [5]	Male; 3 years	Linear calcinosis on the left leg	Malar and heliotrope rash, ulcers (during evolution in indurated areas) and Gottron papules	Proximal (normal muscle enzymes)	Epidermal atrophy, basal vacuolization, chronic perivascular infiltrate and calcium deposition	Colchicine, hydroxychloroquine	Several months (not specified); improvement of rash and general symptoms; residual atrophy within linear lesions
Takahashi et al. [1]	Male; 7 years	Violaceous papules with zosteriform distribution on the back of the right thigh	Heliotrope erythema, Gottron papules, poikiloderma and erythema with silvery white scales	Generalized	Not performed	Prednisolone, methotrexate, topical steroids	Not specified; partial resolution of verruciform papules; improvement of muscle weakness
Lofgren et al.[6]	Male; 11 years	Papules in a linear distribution on the forehead, cheek and nose	Heliotrope rash, malar erythema, Gottron papules, cuticular hypertrophy and telangiectasia	Mild proximal	Not performed	Tacrolimus 0.1% (pre-diagnosis for segmental lesions), prednisone	36 months, resolution of skin and muscle signs and symptoms
Lenormand et al.[7]	Male; 11 years	Blaschko-linear calcinosis on the right thigh	Gottron papules, cuticular necrosis and periungual telangiectasias	None, just myalgias	Not performed	Hydroxychloroquine	Not specified, remission of skin lesions
Makino et al. [8], referred by Happle [4]	Female; 10 years	Linear brownish macules on the right arm and pectoral area, with axillary ulceration	Malar rash	Generalized	Not stated	Not stated	Not stated
Liu et al.[9]	Female; 14 years	Erythematous, scaling papules in a Blaschko-linear distribution on the right abdomen, arm and leg	None	None	Parakeratosis, basal vacuolization and mild perivascular lymphohistiocytic infiltrate	Prednisolone	12 months, no significant improvement of skin lesions, but no new lesions or systemic symptoms
Bulur et al.[2]	Male; 9 years	Erythematous plaque with occasional hypopigmented appearance similar to morphea, extending from the left frontal region to the scalp	Malar erythema, Gottron papules and periungual telangiectasia	None	Vacuolar interface dermatitis, perivascular, perifollicular and perieccrine lymphohistiocytic infiltrates, and dermal mucin	Topical steroids alternating with topical pimecrolimus (pre-diagnosis for segmental lesions), hydroxychloroquine, systemic steroids	Not stated
Topham et al.[10]	Male; 12 years	Thickened, linear, hyperpigmented streak with deep-seated nodules on the leg	Photosensitivity and Gottron papules	None	Vacuolar interface dermatitis on the streak. Deposits of calcium within a nodule	Hydroxychloroquine, topical steroids, topical sodium thiosulfate	Not stated. Regression of the hyperpigmented streak, improvement of dermal calcinosis persisted
Yadav et al.[11]	Male; 5 years	Dusk erythematous plaques with mild atrophy, in a Blaschko-linear distribution over the left half of the body; an ill-defined erythematous firm plaque over the left cheek, in continuity with nonscarring alopecia over the left frontotemporal scalp	Gottron papules and inverse Gottron papules	None	Basal vacuolization, necrotic keratinocytes, pigment incontinence, and perivascular and periadnexal mononuclear infiltrate. Lobar panniculitis is composed of lymphocytes, histiocytes and plasma cells. Thickened basement membrane and increased dermal mucin	Prednisolone, methotrexate, hydroxychloroquine, topical tacrolimus	2 months. 40%-50% improvement in the skin lesions
Alamon-Reig et al.[12]	Female; 7 years	Violaceus lichenoid papules with Blaschkoid distribution on the right forearm	Periungual erythema, dilated capillaries and mild heliotrope rash	Proximal	Vacuolar interface dermatitis and dermal mucin	Methylprednisolone, hydroxychloroquine, methotrexate	Several months (not specified). The cutaneous lesions and systemic symptoms disappeared
Present case	Female; 8 years	Linear, ill-defined, shiny, erythematous plaque on the external surface of the right arm	Malar erythema, heliotrope rash and Gottron papules	Proximal	Focal basal vacuolization, pigment incontinence and perivascular mononuclear infiltrates	Systemic steroids (methylprednisolone, prednisolone), methotrexate. After relapse: methylprednisolone, rituximab, mycophenolate mofetil	48 months. Cutaneous lesions and systemic symptoms disappeared

Regarding classic cutaneous JDM features, 73% had Gottron papules, 45% heliotrope rash, 45% malar erythema, and 36% nailfold changes (e.g., periungual erythema, telangiectasia, and cuticle dystrophy). Muscle weakness was present in 55% of the patients. All segmental lesions had a linear or Blaschkoid distribution. Their topography and morphology are described in Table 2. The median interval from appearance of the segmental lesion to diagnosis was 12 months (range: 1-84).

Biopsy of the segmental lesion was performed in seven patients (64%), all showing features compatible with JDM: basal cell vacuolization, mononuclear perivascular and/or periadnexal dermal infiltrates, interstitial mucin deposition (3/7), and dystrophic calcification (2/7). Overall, 55% of the patients required more than one systemic agent to achieve clinical response. Two patients had received topical treatment for the segmental lesions before the diagnosis of JDM was made.

## Discussion

JDM is an idiopathic inflammatory myopathy of childhood, primarily affecting the skin and muscles. It is characterized by widespread and heterogeneous cutaneous lesions that can significantly impair quality of life if not effectively controlled [13].

Reports of JDM with early, well-localized segmental lesions, such as in our patient, have emerged in recent years [1,2,5-12]. In 2014, Happle named this variant, characterized by a Blaschkoid segmental dermatosis alongside typical JDM features, as a superimposed segmental manifestation of JDM [4]. Similar segmental patterns have been observed in other polygenic skin disorders, including psoriasis, lichen planus, lupus erythematosus, atopic dermatitis, pemphigus vulgaris, vitiligo, graft-versus-host disease, granuloma annulare, and linear drug eruptions [14,15].

SSJDM is believed to result from early developmental genetic mutations that lead to loss of heterozygosity and type 2 cutaneous mosaicism. This produces homozygous skin segments with more severe manifestations adjacent to heterozygous or normal skin [3]. Segmental lesions often appear earlier and are more pronounced than nonsegmental features. However, regarding JDM, the median age at disease onset was nine years, which is slightly higher than the reported age of onset in patients without segmental manifestations [16]. Only one patient had an early onset at the age of three.

The morphology and distribution of segmental lesions are variable, often leading to diagnostic delays of up to 12 months. Based on published clinical descriptions and images, segmental lesions can be grouped into distinct patterns: morpheiform (n = 4), linear calcinosis (n = 2), lichenoid plaques (n = 2), erythematous and scaly plaques (n = 2), and morpheiform plus linear calcinosis (n = 1). Morpheiform lesions (45%) and linear calcinosis (27%) are the most common. Histopathology consistently revealed features compatible with JDM, including basal cell vacuolation, perivascular and periadnexal infiltrates, mucin deposition, and calcinosis, with no evidence of increased collagen deposition or sclerosis, even in morpheiform lesions.

Linear calcinosis, a rare initial finding in JDM, may reflect the severe cutaneous damage associated with loss of heterozygosity in the affected Blaschko-linear segments [5]. SSJDM can mimic other autoimmune connective tissue disorders, leading to possible misdiagnosis of an overlap syndrome. In this regard, Kaur et al. described a patient with linear morphea, overlying lichen sclerosus, calcinosis cutis, and JDM, which could be mistaken for SSJDM. However, histology revealed epidermal atrophy, dermal sclerosis, and calcium deposition, without changes of DM [17]. In SSJDM, the combination of segmental distribution and histological features, vacuolar interface dermatitis and perivascular lymphohistiocytic infiltrates, supports the diagnosis.

In most cases, JDM was not suspected at the onset of segmental lesions, and patients initially received topical therapies, corticosteroids and calcineurin inhibitors, with poor results. Systemic therapy was generally delayed until the development of classic JDM features. This reinforces the clinical importance of recognizing segmental lesions as potential autoimmune indicators: earlier suspicion could reduce the diagnostic delay and allow for timely systemic treatment, potentially improving long-term outcomes.

The number of reported cases remains small, data reporting is inconsistent across publications, and treatment outcomes are frequently incomplete or “not specified.” These gaps limit the strength of conclusions and underscore the need for more systematic reporting of such cases. Nonetheless, the synthesis of available cases highlights consistent findings, most notably that segmental lesions tend to precede systemic disease, and demonstrates the potential value of a structured classification approach to facilitate earlier recognition.

## Conclusions

This report, along with the previously reported cases, highlights the clinical complexity of this rare variant. Segmental lesions consistently preceded systemic manifestations, emphasizing their diagnostic importance. Histopathology of segmental lesions uniformly showed features of JDM, supporting type 2 cutaneous mosaicism as the underlying mechanism. Long-term outcomes varied, emphasizing the need for individualized treatment strategies. Further systematic documentation and larger studies are needed to clarify the pathophysiology, optimize therapeutic approaches, and define the long-term prognosis of this distinctive presentation of JDM.
